# Diarrhea-Predominant Irritable Bowel Syndrome: Medical Management Update

**DOI:** 10.1093/jcag/gwz034

**Published:** 2019-12-03

**Authors:** Christopher N Andrews, Marc Bradette

**Affiliations:** 1 Division of Gastroenterology, University of Calgary, Calgary, Alberta, Canada; 2 Division of Gastroenterology, Centre Hospitalier Universitaire de Québec (CHU), Laval University, Québec, Québec, Canada

**Keywords:** Diarrhea-predominant irritable bowel syndrome, Management, Treatment

## Abstract

Irritable bowel syndrome (IBS) is a prevalent gastrointestinal disorder, which impacts the quality of life, work productivity and social activities of patients. Diarrhea-predominant IBS (IBS-D) is one of several subtypes, and accounts for approximately one third of all cases. Currently available treatments are typically unable to alleviate the cardinal symptoms of IBS-D, including abdominal pain and diarrhea, and a clinical unmet need remains for an effective treatment which simultaneously relieves multiple symptoms. Patients may benefit from a multipronged, individualized approach, including dietary modifications, and psychological and pharmacological therapies. The aim of this review is to provide an update on the available and upcoming treatment options for IBS-D in Canada, with reference to the recently updated Canadian IBS consensus guidelines. Initial treatment approaches include lifestyle modifications, dietary modifications, and non-prescription therapies such as peppermint oil. While some medications such as tricyclic antidepressants are also used to treat IBS-D symptoms, eluxadoline and rifaximin are the only two pharmacological therapies approved for the treatment of IBS-D in Canada. Key clinical trial data for the currently available pharmacological options are presented to provide an overview of the efficacy and safety of these agents

## Irritable Bowel Syndrome Background

Irritable bowel syndrome (IBS) is a common disorder, affecting approximately 11% of the global population (1). It is estimated that approximately five million Canadians may have IBS (2), or 10 to 14% of the population (3). This functional bowel disorder is defined by the presence of recurrent abdominal pain associated with defecation or a change in bowel habits (4). A positive diagnosis of IBS is made based on symptom history using Rome IV criteria (5), with minimal need for diagnostic testing (4). Based on these criteria, patients with IBS can be grouped into one of four subtypes: constipation-predominant IBS, diarrhea-predominant IBS (IBS-D), mixed IBS, where the stool patterns vary from constipation to diarrhea, or unclassified IBS (5). IBS-D accounts for approximately one third of all cases (3). In addition to the cardinal symptoms of IBS-D, including diarrhea and abdominal pain, there are numerous other symptoms including fecal urgency and bloating (4,6–8).

Patients with IBS report significant impact on their work productivity, time management, and participation in social activities due to their symptoms (9). One study found that individuals with IBS in Canada missed the equivalent of 13.8 hours per 40-hour work week due to presenteeism or absenteeism (10). IBS is also associated with comorbid conditions such as anxiety, stress and depression (11), with one study in Canada reporting that 34% of patients with IBS also had comorbid anxiety disorders (12).

The pathogenesis of IBS is thought to be multifactorial, involving visceral hypersensitivity, abnormal gut motility, and dysregulation of the brain–gut axis, among other factors (13). Newer potential etiologic factors include dysbiosis of the gut microflora and small intestinal bacterial overgrowth (SIBO), which can also cause abdominal pain or discomfort, bloating, flatulence and loose stools (14). Treatment with antibiotics has been shown to alleviate symptoms associated with SIBO as well as IBS, including abdominal pain, bloating and diarrhea (15,16). SIBO is often diagnosed using hydrogen breath testing, for which there is currently a lack of standardization in both the substrates used and the preparation and performance of the test, leading to variability in the reported incidence of the condition (16–18). In addition, the concept that SIBO is an etiological factor for IBS is controversial, and the fact that antibiotics can improve SIBO and IBS does not necessarily equate to causation; indeed, antibiotics may be affecting the microflora of the colon rather than the small intestine.

Treatment options are currently limited for patients with IBS-D in Canada, and the heterogeneous nature of this condition presents a challenge in the management of the wide range of observed symptoms; currently available options largely target only one of a wide constellation of symptoms (10). Recently published guidelines from the Canadian Association of Gastroenterology (CAG) highlight that patients with IBS may benefit from a multipronged, individualized approach, including dietary modifications and psychological and pharmacological therapies (19).

The aim of this review is to describe the available and upcoming treatment options for IBS-D in Canada, particularly in relation to the recently updated Canadian IBS consensus guidelines, with a particular focus on eluxadoline and rifaximin, two recent additions to the IBS-D armamentarium.

## Review of Treatment Options

The management of IBS-D symptoms involves lifestyle and diet modifications, over-the-counter therapies, and prescription medications. There is no standard treatment protocol for IBS-D (20), and the limitation of existing therapies is that they often target only one or two IBS symptoms (21). In addition, many treatment regimens are associated with inadequate control of IBS symptoms, which can lead to treatment switching, discontinuation, or use of concomitant therapies (22).

### Lifestyle/Diet Modifications

Lifestyle modifications that may improve IBS symptoms include exercise, stress reduction, and addressing impaired sleep (4). Dietary modifications include the supplementation of soluble fibre as well as restriction of fermentable oligosaccharides, disaccharides, monosaccharides and polyols (FODMAP) (4,19). A recent meta-analysis of seven randomized controlled trials in 397 participants found that a low-FODMAP diet was associated with a reduction in global IBS symptoms compared with a control diet (risk ratio [RR]: 0.69; 95% confidence interval [CI]: 0.54 to 0.88) (23). However, due to the fact that the details of a low-FODMAP diet are readily available on the internet, most of these trials were classified as not blinded (23). In the two studies considered to have adequate levels of blinding, which included 167 patients, the low-FODMAP diet showed no benefit versus an alternative diet (RR: 0.89; 95% CI: 0.68 to 1.17; *P* = 0.84) (19,23–25).

In combination with the low number of participants and heterogeneity in the study design, these factors led to the efficacy of a low-FODMAP diet being designated as having a ‘low quality of evidence’ (23). In addition, a low-FODMAP diet is highly restrictive, and the ability of patients to adhere to such a diet has been shown to impact its effectiveness (20).

### Existing Therapies

#### Psychological Therapies

IBS-D is associated with a high disease burden and low quality of life, which psychological interventions may help to address (26). Referral to psychological treatment may be recommended as part of a multidisciplinary approach to managing IBS symptoms (26). Evidence suggests that psychological therapies, particularly cognitive behavioural therapy and hypnotherapy, can be effective in the management of IBS symptoms. For instance, in a meta-analysis of 15 randomized controlled studies of 1352 patients, psychological therapies (such as cognitive behavioural therapy and stress management) were associated with improvement in IBS symptom severity scales (standardized mean difference [SMD]: −0.618; 95% CI: −0.853 to −0.383), IBS – Quality of Life questionnaire scores (SMD: 0.604; 95% CI: 0.440 to 0.768), and abdominal pain scales compared with controls (SMD: −0.282; 95% CI: −0.562 to −0.001) (27). The CAG has suggested that psychological therapies are a management option, although accessibility to treatment may present challenges for patients (19).

#### Nonprescription Therapies

Nonprescription therapies for the treatment of IBS-D include loperamide, probiotics and peppermint oil. Loperamide is a synthetic peripheral µ-opioid receptor agonist that reduces colonic transit, urgency, and stool consistency in IBS patients (4). In a prospective, double-blind study of patients with IBS (*n* = 69), loperamide was shown to improve stool consistency (by 32%), reduce defecation frequency (by 36%) and reduce the intensity of pain (by 30%) throughout the 5-week study period (28). However, the overall quality of evidence for the use of loperamide in treating IBS has been reported as ‘very low’ by the American College of Gastroenterology (ACG) (29,30). ACG guidelines also suggest that although loperamide is an effective antidiarrheal, there is insufficient evidence to recommend it for the relief of global IBS symptoms (29,30). Similarly, CAG guidelines suggest against offering continuous loperamide to patients with IBS-D (19).

Probiotics are live micro-organisms that can provide health benefits. Across studies, probiotics appear effective in reducing global IBS symptom scores or abdominal pain scores, bloating scores, and flatulence scores (30,31), although the quality of evidence for their use is also considered ‘low’ by the ACG and CAG, particularly due to significant heterogeneity between studies and the use of different probiotics across studies (19,30).

Peppermint oil is a relatively low-cost intervention which has demonstrated consistently favourable results in improving IBS symptoms, and CAG guidelines conditionally suggest offering peppermint oil as a treatment option (19).

### Off-Label Prescription Therapies

A number of prescription therapies are used off-label for the treatment of IBS-D, including bile acid sequestrants and tricyclic antidepressants (21). Bile acid diarrhea may occur in up to one third of patients with IBS-D, and bile acid sequestrants have been reported to improve stool consistency, as demonstrated by data from a pooled analysis of 15 studies comprising 1223 patients with IBS-D (32). Tricyclic antidepressants are also used off-label to treat symptoms of IBS-D, and a recent meta-analysis demonstrated that they may slow gut transit, improve global IBS symptoms and reduce pain (33). CAG guidelines recommend offering low-dose tricyclic antidepressants to improve IBS symptoms (19).

Antispasmodics are used off-label in the treatment of IBS based on the theory that smooth muscle spasms in the gut could contribute to IBS symptoms, particularly abdominal pain or cramps (29). A meta-analysis indicated that as a drug class, treatment with antispasmodics results in significant improvements in IBS symptoms with the number needed to treat of 5 (95% CI: 4 to 9); however, the effect of individual drugs is variable (29,30). In addition, the effects of individual agents are difficult to interpret given the small number of studies completed for the large number of available antispasmodics. Noting very low-quality evidence, CAG guidelines suggest offering certain antispasmodics (e.g., dicyclomine, hyoscine, pinaverium) to patients with IBS (19).

### Approved Prescription Therapies for IBS

Recently, two new treatment options have entered the market for the treatment of IBS-D in Canada. Eluxadoline (Viberzi, Allergan, Parsippany, NJ/Markham, Ontario, Canada) is a novel µ- and κ-opioid receptor agonist and δ-opioid receptor antagonist administered orally twice daily at a dose of 100 mg (34–36). The CAG has made a conditional suggestion in favour of eluxadoline for the treatment of IBS-D symptoms (19).

Rifaximin (Xifaxan, Salix Pharmaceuticals, Bridgewater, NJ/ZAXINE, Lupin Pharma Canada, Montréal, Québec, Canada) is a minimally absorbed broad spectrum antibiotic derived from rifamycin administered orally three times daily at a dose of 550 mg for a total of 14 days (37,38). The recently updated CAG guidelines do not make a recommendation (either for or against) offering one course of rifaximin therapy to patients with IBS-D (19).

### Eluxadoline

#### Phase 2 and 3 Trials

In a Phase 2 trial (NCT01130272), 807 patients meeting Rome III criteria for IBS-D were randomized to receive either placebo or eluxadoline 5, 25, 100 or 200 mg twice daily for 12 weeks (39). A significantly higher proportion of patients treated with eluxadoline 25 mg or 200 mg met the primary composite response criteria at Week 4 and Week 12 compared to placebo (Table 1). After 12 weeks, patients receiving eluxadoline 100 mg or 200 mg had greater improvements in bowel movement frequency, urgency, IBS global symptom scores, IBS severity scores, adequate relief and quality of life scores (39). As the U.S. Food and Drug Administration (FDA) released guidance on outcomes measures for IBS clinical trials after the initiation of this trial, a post-hoc analysis was also completed. A significantly greater proportion of patients in the eluxadoline 100 mg and 200 mg groups were FDA responders after 12 weeks of treatment compared to placebo, defined as ≥30% decrease in their daily worst abdominal pain score and a daily Bristol Stool Form Scale score of <5 or the absence of a bowel movement on ≥50% of days (39).

**Table 1.** Efficacy of eluxadoline in treating IBS-D: summary results from Phase 2, 3 and 4 clinical trials

			Response (patients, %)				
Trial	Treatment (BID)	*n*	Clinical response^a^ Week 4	Clinical response^a^ Week 12	AR^b^ Week 4	AR^b^ Week 8	AR^b^ Week 12
Phase 2 trial NCT01130272 (39)	Placebo	159	5.7	11.3	49.3	53.1	56.8
	ELX 5 mg	105	12.4	8.6	59.1	63.2	67.0
	ELX 25 mg	167	12.0*	13.2	62.4	64.2*	65.9
	ELX 100 mg	163	11.0	20.2*	69.3*	74.9*	79.7*
	ELX 200 mg	160	13.8*	15.0	67.4*	71.5*	75.4*
**Trial**	**Treatment (BID)**	***n***	**CRR** ^**c**^ **Weeks 1–12**	**Abdominal pain** ^**d**^ **Weeks 1–12**	**Stool consistency** ^**e**^ **Weeks 1–12**	**GSS** ^**f**^ **Weeks 1–12**	**AR** ^**g**^ **Weeks 1–12**
IBS-3001 Phase 3 trial NCT01553591 (40)	Placebo	427	17.1	39.6	22.0	28.8	43.8
	ELX 75 mg	426	23.9**	42.4	30.0**	35.1	52.9**
	ELX 100 mg	427	25.1**	43.2	34.3***	34.7	54.2**
IBS-3002 Phase 3 trial NCT01553747 (40)	Placebo	381	16.2	45.3	20.9	29.6	49.2
	ELX 75 mg	382	28.9***	48.0	37.0***	43.6***	60.1**
	ELX 100 mg	382	29.6***	51.0	35.6***	42.4***	58.4**
Pooled data (40)	Placebo	808	16.7	–	–	–	–
	ELX 75 mg	806	26.2***	–	–	–	–
	ELX 100 mg	809	27.0***	–	–	–	–
**Trial**	**Treatment (BID)**	***n***	**CRR** ^**c**^ **Weeks 1–4**	**CRR** ^**c,h**^ **Weeks 9–12**	**CRR** ^**c,h**^ **Weeks 21–24**	**CRR** ^**c,h**^ **Weeks 1–12**	**CRR** ^**c,h**^ **Weeks 1–26**
Post-hoc analysis of IBS-3001 and IBS-3002 (41)	Placebo	809 101^h^	12.5	68.3	49.5	77.2	66.3
	ELX 75 mg	808 184^h^	22.8**	72.8	63.0	81.5	73.9
	ELX 100 mg	806 198^h^	24.6**	71.7	57.1	77.8	70.7
**Trial**	**Treatment (BID)**	***n***	**CRR,** ^**c**^ **ISC Weeks 1–12**	**CRR,** ^**c**^ **ASC Weeks 1–12**	**CRR,** ^**c**^ **ISC Weeks 1–26**	**CRR,** ^**c**^ **ASC Weeks 1–26**	
Post-hoc analysis of IBS-3001 and IBS-3002 in patients who reported prior loperamide use (42)	Placebo	ISC (*n* = 166) ASC (*n* = 116)	12.7	25.0	17.5	26.7	
	ELX 75 mg	ISC (*n* = 198) ASC (*n* = 96)	26.3***	37.5	26.8*	36.5	
	ELX 100 mg	ISC (*n* = 174) ASC (*n* = 122)	27.0***	41.8**	31.6**	44.3**	
**Trial**	**Treatment (BID)**	***n***	**CRR** ^**i**^ **Weeks 1–12**	**Abdominal pain** ^**j**^ **Weeks 1–12**	**Stool consistency** ^**k**^ **Weeks 1–12**		
Phase 4 trial NCT02959983 (43)	Placebo	174	10.3	31.0	16.7		
	ELX 100 mg	172	22.7**	43.6*	27.9**		

*P < 0.05; **P ≤ 0.01; ***P ≤ 0.001 vs. placebo. ^a^Defined as a decrease in mean daily WAP scores from baseline by ≥30% and at least 2 points on a scale from 0 to 10 and a daily BSFS of 3 or 4 on ≥66% of daily diary entries within that week. ^b^Defined as a positive answer to the question, ‘Over the past week have you had adequate relief of your IBS symptoms?’ on a monthly basis. ^c^Defined as a ≥30% reduction from the average baseline score for WAP on ≥50% of days and, on the same days, a stool consistency score of <5; if the patient did not have a bowel movement, an improvement of ≥30% in the WAP score was sufficient for a response on that day. ^d^Defined as a ≥30% reduction from baseline in the WAP score on ≥50% of days in patients with ≥60 days of electronic diary entries from Weeks 1–12. ^e^Defined as a stool consistency score of <5, or the absence of a bowel movement if accompanied by a ≥30% improvement in the WAP score, on ≥50% of days in patients with ≥60 days of electronic diary entries from Weeks 1–12. ^f^Defined as a score of 0 or 1 on a scale of 0–4, where 0 indicates no symptoms and 4 indicates very severe symptoms, or an improvement of ≥2 from baseline, on ≥50% of days in patients with ≥60 days of electronic diary entries from Weeks 1–12. ^g^Defined as a positive response to the question, ‘Over the past week, have you had adequate relief of your IBS symptoms?’ in patients with ≥6 weeks of data from the 12-week interval (Weeks 1–12). ^h^Of the patients who were composite responders at Weeks 1–4. ^i^Defined as the proportion of patients who met daily composite response criteria (≥40% WAP improvement and <5 BSFS) for ≥50% of treatment days, and recorded ≥60 days of diary entries over the 12-week period. ^j^Defined as the proportion of patients who met daily pain response (WAP score improvement by ≥40% in the preceding 24 hours) for ≥50% of days, and recorded ≥60 days of diary entries for the full 12 weeks. ^k^Defined as the proportion of stool consistency responders (patients who met the daily stool consistency of <5 BSFS) for ≥50% of days with ≥60 days of diary entries for the full 12 weeks.

AR, adequate relief; ASC, adequate symptom control with prior loperamide; BID, twice daily; BSFS, Bristol Stool Form Scale; CRR, composite response rate; ELX, eluxadoline; GSS, global symptom score; IBS-D, diarrhea-predominant irritable bowel syndrome; ISC, inadequate symptom control with prior loperamide; WAP, worst abdominal pain.

**Figure 1.**
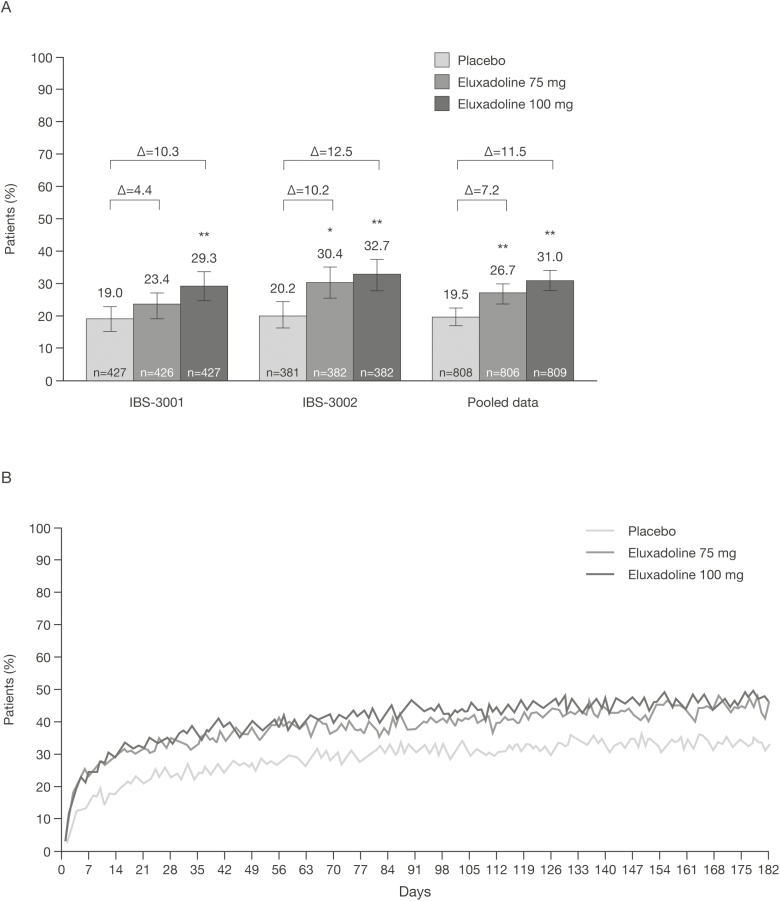
Eluxadoline efficacy in Phase 3 trials. Proportions of patients achieving composite response^a^ after 26 weeks of treatment in the Phase 3 clinical trials (individual trials and pooled data) after 26 weeks of treatment (A) and the proportion of composite responders^a^ over time in the pooled trials (B). *P < 0.05; **P < 0.001 vs. placebo. ^a^Defined as a ≥30% reduction from the average baseline score for worst abdominal pain on ≥50% of days and, on the same days, a stool consistency score of <5. If the patient did not have a bowel movement, an improvement of ≥30% in the worst abdominal pain score was sufficient for a response on that day. Figure 1A and B reproduced from Lembo et al. (40). in the *New England Journal of Medicine* by permission of Massachusetts Medical Society. Copyright ©2016 Massachusetts Medical Society.

Two randomized, double-blind, placebo-controlled Phase 3 trials enrolled 2428 patients with IBS-D who met Rome III criteria (IBS-3001, NCT01553591 [52 weeks] and IBS-3002, NCT01553747 [26 weeks]) to receive placebo or eluxadoline 75 or 100 mg twice daily (40). The primary efficacy endpoint of both trials was the FDA composite response, consisting of a simultaneous improvement in both abdominal pain and stool consistency, evaluated at 12 and 26 weeks of treatment. Pooled data from the two clinical trials demonstrated that a significantly higher proportion of patients in the eluxadoline group were composite responders as compared to the placebo group (Figure 1A; Table 1). Improvements in symptoms, as assessed via the composite response, were evident within the first week of treatment in patients receiving eluxadoline and were maintained throughout the 26-week treatment period (Figure 1B) (40).

A significantly greater proportion of patients receiving either dose of eluxadoline were stool consistency responders at Week 12 (Table 1) (40). Eluxadoline was also effective in terms of number of urgency-free days, frequency, and bloating, with a significant reduction observed in these outcomes compared to placebo in the pooled analysis. A greater proportion of patients receiving eluxadoline were IBS global symptom responders and a significantly greater proportion of patients were adequate relief responders in both Phase 3 clinical trials (Table 1). Eluxadoline demonstrated effectiveness from the first week of treatment; a post-hoc analysis indicated that an early clinical response to eluxadoline was shown to be associated with sustained response for up to 6 months (Table 1) (41).

In the two Phase 3 trials, 36.0% reported prior loperamide use (40) and of those, 61.8% reported inadequate symptom control (42). A post-hoc analysis assessed response to eluxadoline treatment in these patients (42); a greater proportion treated with eluxadoline were composite responders over 26 weeks as compared to those treated with placebo (Table 1). Efficacy was comparable irrespective of the use of loperamide as a rescue medication during the trial period (42).

### Phase 4 Trial

The efficacy and safety of eluxadoline was evaluated in a Phase 4 trial (NCT02959983) in IBS-D patients who reported inadequate symptom control with prior loperamide (43). A significantly greater proportion of eluxadoline patients achieved the primary composite responder endpoint compared to placebo (22.7% versus 10.3%; *P* = 0.002), which was also reflected in the component endpoints (Table 1).

### Safety

Eluxadoline was well tolerated in clinical trials. The most common adverse events (AEs) were constipation, abdominal pain and nausea (39,40,44) (Table 2). In the pooled Phase 2 and 3 trials, pancreatitis was the most commonly reported serious AE (SAE) among eluxadoline-treated patients; however, the overall incidence was low (0.4% of eluxadoline-treated patients). Ten events consistent with sphincter of Oddi spasm were reported in patients receiving eluxadoline, and seven events of pancreatitis were reported, all of which were defined as mild, and all patients discontinued treatment at event onset (44). No cases of sphincter of Oddi spasm or pancreatitis were reported in the Phase 4 trial (43). Eluxadoline is contraindicated in patients without a gallbladder, or patients who consume >3 alcoholic beverages a day, due to an increased risk of developing pancreatitis and/or sphincter of Oddi spasm (45,46). Patients should avoid excessive alcohol consumption while taking eluxadoline.

**Table 2.** Safety overview of the eluxadoline clinical trials: pooled Phase 2 and Phase 3 data (44)

AEs, *n* (%)	Placebo BID, *n* = 975	ELX 75 mg BID,^a^*n* = 807	ELX 100 mg BID, *n* = 1032
Patients with ≥1 AE	533 (54.7)	486 (60.2)	575 (55.7)
Events	1573	1556	1804
Patients with ≥1 SAE	25 (2.6)	34 (4.2)	41 (4.0)
Events	28	40	65
Deaths	0	0	0
Patients with SOS	0	2 (0.2)	8 (0.8)
Patients with pancreatitis^b^	0	3 (0.4)	4 (0.4)
Any AE leading to discontinuation^c^	42 (4.3)	67 (8.3)	80 (7.8)
Constipation	3 (0.3)	9 (1.1)	15 (1.5)
Abdominal pain	3 (0.3)	9 (1.1)	11 (1.1)
Nausea	4 (0.4)	5 (0.6)	0
Abdominal distension	1 (0.1)	2 (0.2)	5 (0.5)
Abdominal pain upper	0	3 (0.4)	4 (0.4)
Pancreatitis^d^	0	3 (0.4)	3 (0.3)
Headache	1 (0.1)	3 (0.4)	1 (0.1)
Diarrhea	3 (0.3)	1 (0.1)	0
AEs reported in ≥2% of any treatment group			
Constipation	24 (2.5)	60 (7.4)	84 (8.1)
Nausea	49 (5.0)	65 (8.1)	73 (7.1)
URTI	38 (3.9)	27 (3.3)	53 (5.1)
Abdominal pain	25 (2.6)	33 (4.1)	47 (4.6)
Headache	44 (4.5)	32 (4.0)	44 (4.3)
Vomiting	12 (1.2)	32 (4.0)	43 (4.2)
Dizziness	21 (2.2)	21 (2.6)	33 (3.2)
Flatulence	17 (1.7)	21 (2.6)	33 (3.2)
Nasopharyngitis	33 (3.4)	33 (4.1)	31 (3.0)
Bronchitis	21 (2.2)	26 (3.2)	30 (2.9)
Abdominal distension	15 (1.5)	21 (2.6)	28 (2.7)
Sinusitis	35 (3.6)	27 (3.3)	27 (2.6)
ALT increased	14 (1.4)	17 (2.1)	26 (2.5)
Fatigue	23 (2.4)	21 (2.6)	20 (1.9)
UTI	17 (1.7)	17 (2.1)	18 (1.7)
Gastroenteritis viral	18 (1.8)	22 (2.7)	14 (1.4)
Hypertension	16 (1.6)	20 (2.5)	14 (1.4)

^a^ELX 75 mg was not evaluated in the Phase 2 trial. ^b^Includes one event reported by the investigator as pancreatitis but adjudicated as not meeting Atlanta criteria for pancreatitis. ^c^Occurring in ≥3 patients in any treatment group. ^d^Includes all acute pancreatitis and pancreatitis events; one pancreatitis SAE was reported (ELX 100 mg group) but did not prompt discontinuation as the patient had been off trial drug for 2 weeks before event onset.

AE, adverse event; ALT, alanine aminotransferase; BID, twice daily; ELX, eluxadoline; SAE, serious adverse event; SOS, sphincter of Oddi spasm; URTI, upper respiratory tract infection; UTI, urinary tract infection.

Table 2 reproduced from Cash et al. (44). in *Am J Gastroenterol.* Available at https://www.ncbi.nlm.nih.gov/pmc/articles/PMC5318664/. Copyright ©2016 The Authors https://creativecommons.org/licenses/by-nc-nd/4.0/legalcode.

Eluxadoline has been reported to interact with cyclosporine, strong CYP inhibitors, and drugs that cause constipation (34,36). While eluxadoline is listed as a controlled substance due to its interaction with opioid receptors (34,36), a post-hoc analysis of Phase 2 and Phase 3 clinical trial data demonstrated a lack of abuse potential with eluxadoline treatment (47). The incidence of AEs potentially related to abuse (i.e., dizziness, fatigue, anxiety, etc.) was similar between the placebo, eluxadoline 75 mg, and eluxadoline 100 mg treatment groups, and using the subjective opioid withdrawal scale, there was minimal evidence of withdrawal symptoms and no significant difference between the treatment groups in terms of withdrawal scores (47). Intranasal and oral eluxadoline abuse potential was also evaluated in healthy volunteers and demonstrated lower abuse potential than oxycodone (48).

### Rifaximin

#### Phase 2 and 3 Trials

In a Phase 2 clinical trial (NCT00259155), 87 patients who met Rome I criteria for IBS were enrolled and randomized to receive rifaximin 400 mg three times daily or placebo for 10 days and were followed for 10 weeks post-treatment (49). Rifaximin resulted in greater improvements in IBS symptoms as compared to placebo over the 10 weeks of follow-up, with rifaximin-treated patients experiencing 36.4% improvement compared with 21.0% improvement in placebo-treated patients (*P* = 0.02) (49).

**Figure 2.**
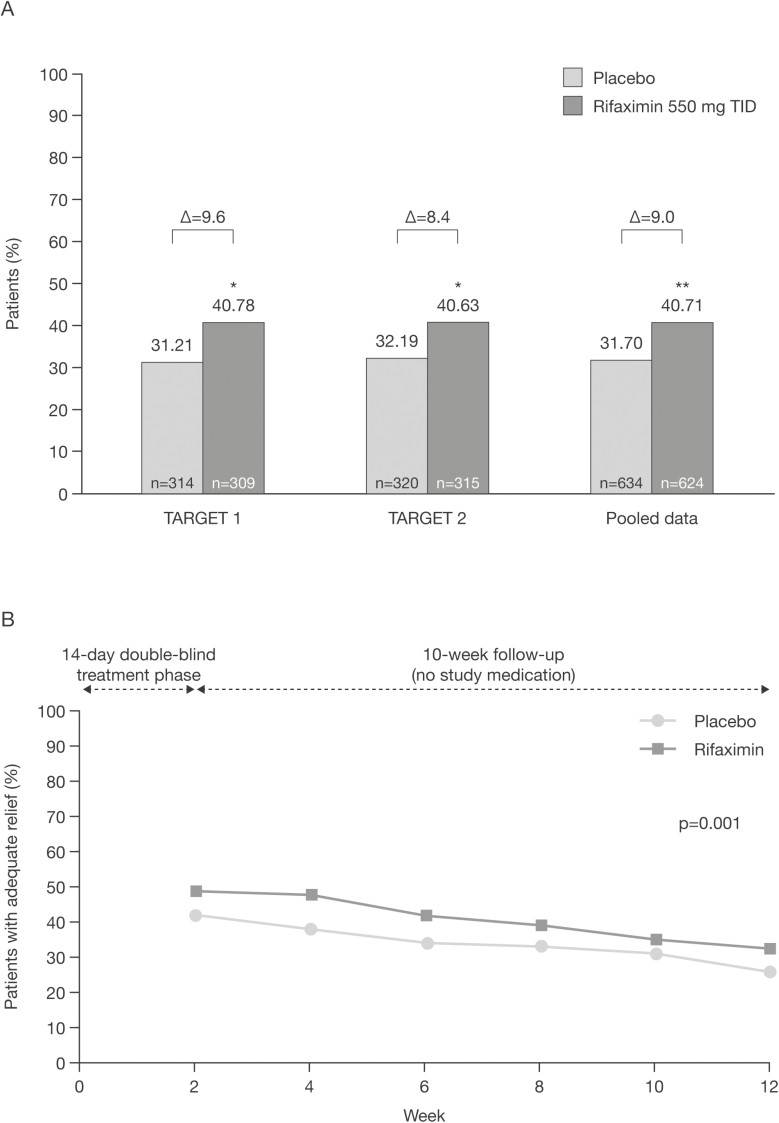
Rifaximin efficacy in Phase 3 trials. The proportion of patients achieving adequate relief of global IBS symptoms^a^ from Weeks 3 to 6 (A) and the proportion of patients achieving adequate relief of global IBS symptoms^a^ from the pooled trials over time (B). *P < 0.05; **P < 0.001 vs. placebo. ^a^Defined as a positive response to the question, ‘In regards to all your symptoms of IBS, as compared with the way you felt before you started the study medication, have you, in the past 7 days, had adequate relief of your IBS symptoms?’ for ≥2 of the 4 weeks during Weeks 3–6. IBS, irritable bowel syndrome; TID, three times daily. Figure 2B reproduced from Pimentel et al. (50). in the *New England Journal of Medicine* by permission of Massachusetts Medical Society. Copyright ©2011 Massachusetts Medical Society.

In two double-blind, placebo-controlled Phase 3 trials (NCT00731679, NCT00724126), 1260 patients with IBS without constipation were randomly assigned to rifaximin 550 mg or placebo three times daily for 2 weeks, and were followed for 10 subsequent weeks (50). The pooled data from the two studies demonstrated that significantly more patients in the rifaximin group had adequate relief of global IBS symptoms during the first 4 weeks following treatment than the placebo group, and maintained from 2 to 12 weeks post-treatment (Figure 2A and B). In addition, significantly more patients in the rifaximin group had adequate relief of bloating, achieved abdominal pain response and stool consistency response, and achieved the composite abdominal pain and stool consistency response than in the placebo group in the pooled analysis (Table 3) (50).

**Table 3.** Rifaximin efficacy in the treatment of IBS-D: summary data from two Phase 3 clinical trials

Response (patients, %)								
Trial	Treatment (TID)	*N*	Weekly IBS- related bloating^a^	Daily global IBS symptoms^b^	Daily IBS- related bloating^c^	Daily abdominal pain and stool consistency^d^	Daily abdominal pain^e^	Daily stool consistency^f^
TARGET 1 NCT00731679 (50)	Placebo	314	28.7	30.6	32.5	38.5	42.0	67.5
	Rifaximin 550 mg	309	39.5*	42.7**	39.2*	46.6*	51.5*	79.0*
TARGET 2 NCT00724126 (50)	Placebo	320	31.9	28.4	30.9	36.3	43.1	64.4
	Rifaximin 550 mg	315	41.0*	37.8*	43.5**	46.7*	52.4*	74.0*
Pooled analysis (50)	Placebo	634	30.3	29.5	31.7	37.4	42.6	65.9
	Rifaximin 550 mg	624	40.2**	40.2**	41.3**	46.6**	51.9**	76.4**
**Trial**	**Treatment (TID)**	***n***	**CR** ^**g**^	**Abdominal pain** ^**h**^	**Stool consistency** ^**i**^	**Prevention of recurrence** ^**j**^	**Durable response** ^**k**^	**Bloating** ^**l**^
TARGET 3 NCT01543178 (51)	Placebo	308	31.5	42.2	50.0	7.1	11.7	41.2
	Rifaximin 550 mg	328	38.1*	50.6*	51.8	13.2*	17.1*	46.6

*P ≤ 0.05; **P ≤ 0.001 vs. placebo.

^a–h^All endpoints are assessed during Weeks 3–6 (the first 4 weeks following treatment). ^g–l^Endpoints are assessed during the first 4 weeks following the first repeat treatment. ^a^Defined as a positive response to the question, ‘In regards to your symptoms of bloating, as compared with the way you felt before you started the study medication, have you, in the past 7 days, had adequate relief of your IBS symptom of bloating?’ ^b^Defined as a score of 0 (not at all) or 1 (hardly) for ≥50% of days in a given week for their IBS symptoms. ^c^Defined as a score of 0 (not at all) or 1 (hardly) for ≥50% of days in a given week for their IBS symptom of bloating. ^d^Defined as a decrease of ≥30% from baseline in weekly mean ratings of IBS-related abdominal pain or discomfort and a weekly mean stool consistency score of <4 (with 4 indicating loose stools and lower scores indicating more formed stools). ^e^Defined as a decrease of ≥30% from baseline in weekly mean ratings of IBS-related abdominal pain or discomfort. ^f^Defined as a weekly main stool consistency score of <4 (with 4 indicating loose stools and lower scores indicating more formed stools). ^g^Defined as a decrease in abdominal pain of ≥30% from baseline and a decrease in number of days per week with BSFS type 6 or 7 stool of ≥50% from baseline, for ≥2 weeks during a 4-week post-treatment period after first repeat treatment. ^h^Defined as a decrease in abdominal pain of ≥30% from baseline for ≥2 weeks during a 4-week post-treatment period after first repeat treatment. ^i^Defined as a decrease in number of days per week with BSFS type 6 or 7 stool of ≥50% from baseline for ≥2 weeks during a 4-week post-treatment period. ^j^Defined as adequate relief in both abdominal pain and stool consistency during Weeks 1–4 following treatment, with no recurrence through the 6-week treatment-free repeat treatment observation phase and the second 6-week repeat treatment phase. ^k^Defined as adequate relief in both abdominal pain and stool consistency during Weeks 1–4 following treatment with no recurrence through the 6-week treatment-free repeat treatment observation phase. ^l^Defined as the percentage of patients with ≥1-point decrease (on a scale of 0–6, where 0 indicates not at all and 6 indicates a very great deal) from baseline in weekly average bloating score for ≥2 weeks during the 4-week primary evaluation period.

BSFS, Bristol Stool Form Scale; CR, composite response; IBS, irritable bowel syndrome; IBS-D, diarrhea-predominant irritable bowel syndrome; TID, three times daily.

In order to investigate the need for rifaximin re-treatment for long-term response, a randomized, placebo-controlled, 51-week Phase 3 trial (NCT01543178) enrolled patients with IBS without constipation (51). The trial consisted of a 2-week open-label treatment phase (rifaximin 500 mg three times daily) followed by a 4-week assessment phase, and efficacy was evaluated in 2438 patients. Patients who initially responded and subsequently experienced a relapse in IBS-D symptoms entered into the double-blind treatment phase. During the observation phase, 692 patients experienced a relapse; subsequently, 636 were randomized to double-blind repeat treatment. Patients were randomized to receive two repeat treatment courses of rifaximin 550 mg or placebo three times daily for 14 days, and were followed for 4 weeks after each treatment, with a treatment-free 6-week observation phase between the rounds of treatment. The percentage of responders during the double-blind treatment phase was significantly greater with rifaximin than with placebo (Table 3). The proportions of abdominal pain responders, prevention of recurrence responders, and durable response responders were also significantly greater with rifaximin treatment than with placebo (Table 3).

### Safety

The AE rates observed during the clinical trials were low overall and were similar between the rifaximin and placebo groups (51). The most common AEs with rifaximin treatment included headache, upper respiratory tract infection and nausea (Table 4) (50,51). Incidences of drug-related AEs, SAEs, and infection-associated AEs were similar between the placebo and rifaximin groups in the pooled Phase 2 and Phase 3 clinical trial data (52). The incidence and types of AEs were similar with repeat rifaximin treatment (51). Rifaximin demonstrates very minimal drug–drug interactions, and is only known to interact significantly with cyclosporine (37,38).

**Table 4.** Safety overview of the rifaximin clinical trials

AE, *n* (%)	Placebo *n* = 829	All pooled rifaximin *n* = 1103
Any AE	436 (52.6)	579 (52.5)
Specific AE in ≥2% of patients^a^		
Headache	51 (6.2)	59 (5.3)
URTI	47 (5.7)	50 (4.5)
Nausea	31 (3.7)	48 (4.4)
Abdominal pain	39 (4.7)	41 (3.7)
Diarrhea	26 (3.1)	37 (3.4)
UTI	18 (2.2)	37 (3.4)
Nasopharyngitis	39 (4.7)	26 (2.4)
Sinusitis	23 (2.8)	24 (2.2)
Vomiting	12 (1.4)	22 (2.0)
Back pain	19 (2.3)	22 (2.0)
AE severity^b^		
Mild	169 (20.4)	268 (24.3)
Moderate	214 (25.8)	246 (22.3)
Severe	53 (6.4)	63 (5.7)
Drug-related AEs	89 (10.7)	134 (12.1)
SAEs		
Any SAE	18 (2.2)	16 (1.5)
Drug-related SAE	2 (0.2)	1 (0.1)
Deaths	0	0
AEs resulting in study discontinuation		
Any AE	14 (1.7)	22 (2.0)
Drug-related AE	7 (0.8)	9 (0.8)

Adapted from Schoenfeld et al (52). in *Alimentary Pharmacology & Therapeutics*, by permission of John Wiley & Sons Ltd. Copyright ©2014 The Authors.

^a^Occurring in ≥2% of patients in either rifaximin group or in the placebo group. ^b^Data not available for two AEs in the rifaximin group.

AE, adverse event; SAE, serious adverse event; URTI, upper respiratory tract infection; UTI, urinary tract infection.

### Antibiotic Resistance Considerations

Rifaximin is administered orally three times daily at a dose of 550 mg for a total of 14 days, with up to two re-treatments for patients who experience symptom recurrences (37,38). In the Phase 2 and Phase 3 clinical trials, the effect of rifaximin seemed to decrease over time (Figure 2B), and patients experienced a relapse in occurrence of IBS symptoms, resulting in administration of repeat courses of rifaximin (51,53). Despite the concerns surrounding repeat treatment with antibiotics, short-term rifaximin treatment has not shown any association with clinically relevant antibiotic resistance (53). In post-hoc analyses of Phase 3 trials, it was demonstrated that *Clostridium difficile* was highly susceptible to rifaximin. In addition, rifaximin exposure was not associated with long-term cross resistance to rifampin or tested nonrifamycin antibiotics (53).

While data indicate that rifaximin is effective in patients with IBS-D and mixed IBS, the mechanism of action for its benefits is largely unknown and warrants further investigation (54). Although short courses of rifaximin have been shown not to result in antibiotic resistance, rifamycins are important for the treatment of serious infections, and the use of an antibiotic to treat a common disorder without understanding its mechanism of action raises a concern (53). With data showing that the durability of the effect of rifaximin decreases over time, the possibility of multiple re-treatments with an antibiotic that has a diminishing effect raises further concerns regarding antibiotic resistance (51).

## Conclusions

IBS is a prevalent gastrointestinal disorder, which significantly impacts patients’ quality of life. Presentation of patients with IBS is common in gastroenterology clinics and practices, and physicians need to be aware that safe and effective treatments exist and can help their patients. The heterogeneous presentation and multifactorial pathogenesis of IBS-D require an individualized approach to the management of IBS-D symptoms. Eluxadoline and rifaximin are two novel treatments for adults with IBS-D that show promising efficacy and safety for this disorder with a high burden of illness. Real-world studies are warranted to provide information on which patients would most benefit from various treatment regimens.
